# On the relative importance of space and environment in farmland bird community assembly

**DOI:** 10.1371/journal.pone.0213360

**Published:** 2019-03-11

**Authors:** Laura Henckel, Christine N. Meynard, Vincent Devictor, Nicolas Mouquet, Vincent Bretagnolle

**Affiliations:** 1 Centre d'Etudes Biologiques de Chizé (CEBC), UMR 7372 CNRS & Université de La Rochelle, Beauvoir sur Niort, France; 2 CBGP, INRA, CIRAD, IRD, Montpellier SupAgro, Univ Montpellier, Montpellier, France; 3 Institut des Sciences de l'Evolution, Université de Montpellier, CNRS, IRD, EPHE, Place Eugène Bataillon, Montpellier Cedex 05, France; 4 MARBEC, Univ Montpellier, CNRS, Ifremer, IRD, Montpellier, France; 5 LTSER “Zone Atelier Plaine & Val de Sèvre”, Beauvoir sur Niort, France; Irstea, FRANCE

## Abstract

The relative contribution of ecological processes in shaping metacommunity dynamics in heavily managed landscapes is still unclear. Here we used two complementary approaches to disentangle the role of environment and spatial effect in farmland bird community assembly in an intensive agro-ecosystem. We hypothesized that the interaction between habitat patches and dispersal should play a major role in such unstable and unpredictable environments. First, we used a metacommunity patterns analysis to characterize species co-occurrences and identify the main drivers of community assembly; secondly, variation partitioning was used to disentangle environmental and geographical factors (such as dispersal limitation) on community structure and composition. We used high spatial resolution data on bird community structure and composition distributed among 260 plots in an agricultural landscape. Species were partitioned into functional classes, and point count stations were classified according to landscape characteristics before applying metacommunity and partitioning analyses within each. Overall we could explain around 20% of the variance in species composition in our system, revealing that stochasticity remains very important at this scale. However, this proportion varies depending on the scale of analysis, and reveals potentially important contributions of environmental filtering and dispersal. These conclusions are further reinforced when the analysis was deconstructed by bird functional classes or by landscape habitat classes, underlining trait-related filters, thus reinforcing the idea that wooded areas in these agroecosystems may represent important sources for a specific group of bird species. Our analysis shows that deconstructing the species assemblages into separate functional groups and types of landscapes, along with a combination of analysis strategies, can help in understanding the mechanisms driving community assembly.

## Introduction

Understanding the mechanisms involved in community assembly is a major challenge for ecologists. Meta-community theory suggests that the composition of a community results from four kinds of mechanisms: biotic interactions, environmental filtering, dispersal and demographic stochasticity [1–3]. Since ecological parameters (e.g., demography or dispersal) are often extremely difficult to estimate, indirect methods consisting of analyzing spatial patterns of species distributions have often been used to disentangle these mechanisms in the field [4]. However, interpreting the results of these analyses is still challenging because several mechanisms can lead to the same spatial distribution patterns [5]. For instance, some biotic interactions (such as facilitation) may lead to aggregation of individuals, resulting in a positive spatial autocorrelation; on the contrary, competition may lead to overdispersion between closely related species, which would exclude each other in close proximity [6]. Other mechanisms that can generate spatial autocorrelation include population dynamics (reproduction and mortality), local dispersal [7], and environmental filtering [8]. When environmental variables are themselves spatially structured, and species with similar functional traits are likely to be found in similar habitats, disentangling the share of exogenous autocorrelation (spatial auto-correlation due to the spatial distribution of habitats) and endogenous autocorrelation (due to population dynamic and dispersal) is often difficult [7]. Even so, recent studies have suggested that models including spatial autocorrelation due to dispersal, as well as stochastic events of colonization and extinction, can better represent metacommunity structure and species distribution ranges as compared to models based on environmental variation only [9,10]. Hence, the study of the spatial structure of community composition is useful in disentangling community drivers, but it is not sufficient to distinguish between mechanisms driving community assembly.

Adding to this difficulty, there is an increasing recognition that all these processes (dispersal, demography, biological interactions and environmental filtering) seem to be complementary rather than exclusive [11,12], and their relative contribution varies between communities and ecosystems, and across spatial scales [13,14]. For example, environmental filtering may generally account for species distributions at large scales, but this effect varies strongly between taxa and ecosystems [14]. In addition, spatial or temporal environmental heterogeneity [9,15], as well as dispersal under certain conditions, may allow species coexistence despite environmental filtering [10]. These findings point to the need to consider the particularities of scales and taxa when trying to understand community assembly.

One complementary solution to link empirical observations to mechanisms in community ecology has involved the incorporation of functional traits, which can help to distinguish different types of mechanisms related to competition and environmental filtering among others [9,15]. Indeed, since ecological communities can host a large number of species with different life history traits, partitioning coexistence using global statistical partitioning might lead to results that are difficult to interpret. An appealing solution is to divide communities into smaller clusters of ecologically similar species, using a deconstruction approach [16–18]. The deconstruction approach is meant to identify key groups of species that respond similarly to major variables, and therefore allow a better understanding of the variability observed when all species are considered together. This would ensure that the observed spatial patterns are the result of similar species responding to similar drivers, as opposed to different species having opposing responses that will blur the global patterns and impede the finding of clear explanations. While studies that combine different community assembly mechanisms are becoming more common, (e.g., [19,20]), very few have tried to disentangle the relative contribution of these processes depending on habitat characteristics or species ecology, and most have been conducted in aquatic ecosystems, while studies on terrestrial ecosystems focus mainly on plants [14]. Largely missing from this literature is the study of animal communities in terrestrial ecosystems, especially in human-dominated or managed ones [14].

Here we applied a methodology to disentangle community assembly processes including a clustering by habitat and species groups. This study focuses on highly disturbed ecosystems that are managed for resource production (farmland landscapes), in which species may further depend on local source-sink dynamics to subsist in an environment that is highly variable and constantly perturbed (e.g., seasonality of farming practices, crop rotation). Our case study is a fairly rich farmland bird metacommunity of 40 species, in a cereal agro-ecosystem in the west of France. This biological system is characterized by a high functional diversity of species coexisting in highly heterogeneous and changing habitats. Intensive sampling during 5 years allowed fine scale analysis of species distributions. Contrary to the majority of studies that analyzed species distribution patterns at broad scales [21–23], we used a scale of analysis that matches the size of the breeding territory of most farmland bird species [24–26], as well as complementary methods allowing testing different processes simultaneously.

Our methodology involves a functional and landscape deconstruction strategy, along with spatial and partial regression analyses, to understand the relative contributions of environmental filtering, dispersal and neutral processes (demographic stochasticity). We used three complementary approaches. First, metacommunity patterns sensu Presley et al. [5,27] were analyzed to characterize the global patterns of co-occurrence and identify the most important environmental gradients and coherent groups of species. This approach allows distinguishing patterns resulting from species sorting, biotic interactions (competition) and random assembly processes [19]. Second, a variation partitioning approach was used to disentangle the relative contributions of environment (exogenous autocorrelation) and neutral processes (endogenous autocorrelation) in community composition, see [19]. Third, the two approaches were combined with a deconstruction approach (by species group and landscape type) to see if different groups of species or landscapes presented a different influence of community assembly drivers. Finally, as these processes can be highly scale-dependent, analyses were conducted at various spatial grains.

## Materials and methods

### Study area

**Fig 1:**
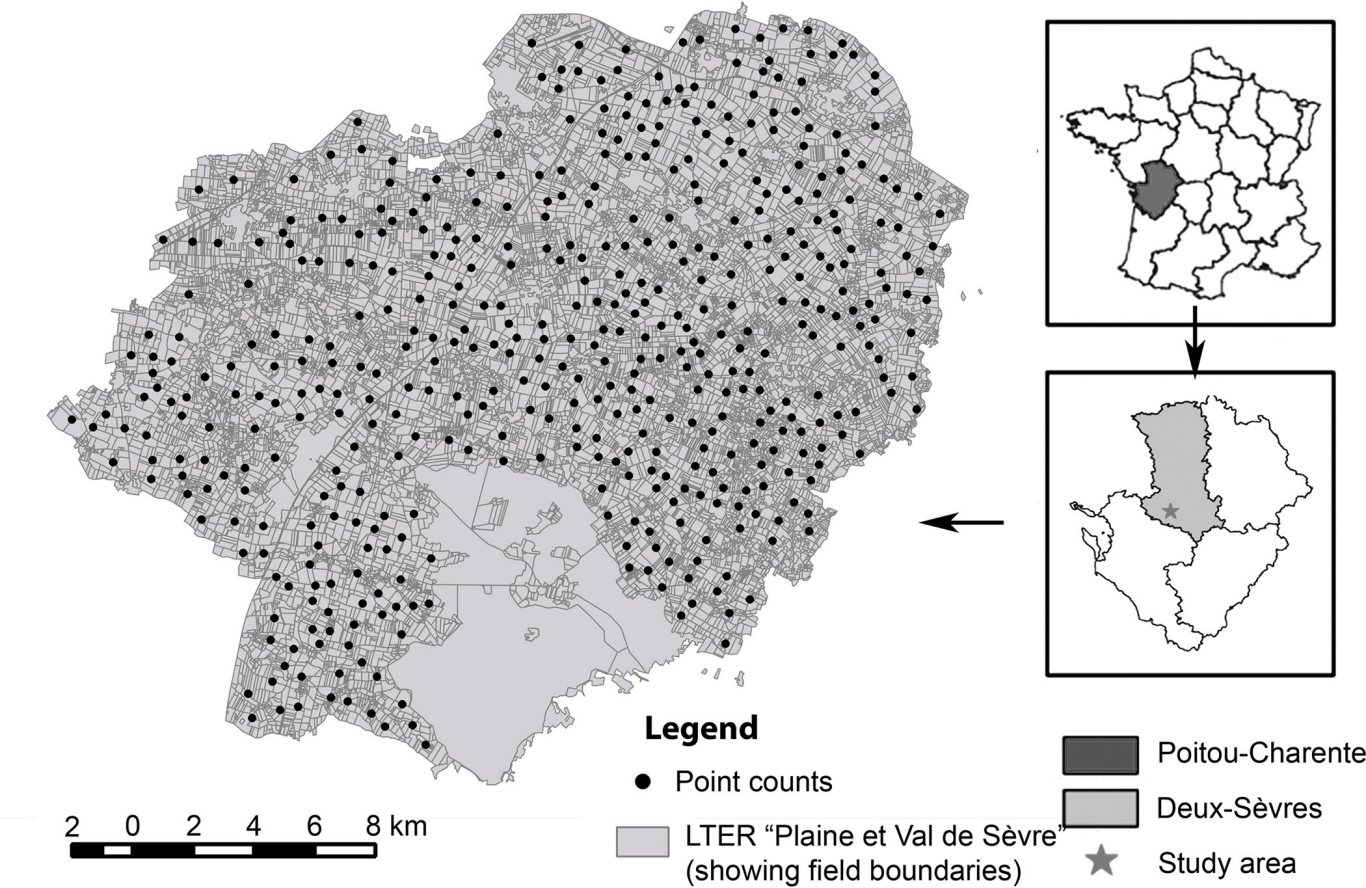
Location of the LTER Plaine et Val de Sèvre (study area) and positions of the 260 point count stations. These stations are distributed fairly evenly over the whole study area.

The study site was the Zone Atelier Plaine & Val de Sèvre, an area of 429 km^2^ which is part of the French Long Term Ecological Research network, see [28], located in central western France, in the Poitou-Charentes Region (France; 46.23°N, 0.41°W, Fig 1). It is an intensive farming system, where cereal crops are dominant (e.g., on average during the 5-year period included in this study, 36.8% of the surface was occupied by winter cereals, with 33.3% represented by wheat and 3.1% by barley). Other main crops include rapeseed (8.7%), maize (8.6%), sunflower (10.7%), grassland (7.8%) and alfalfa (3.9%). The mean field size (11,000 fields) was 3.7 ha.

### Point counts

Breeding birds were surveyed on a total of 260 locations (approximately one per square kilometer) using point counts spread over the whole study area (Fig 1). For each point count, we recorded all birds seen or heard during 5 minutes in a 200 meter radius. All individuals were located on a map by the observer to avoid double counting. The same point counts were surveyed twice during the breeding season (mean range date 22/04-16/05 for session 1 and 24/05–28/06 for session 2), and every year from 2009 to 2013. These survey dates allow to take into account both early and late migrants. Five minute point counts are appropriate to have a good estimate of the community composition [29,30], allowing to cover more points while limiting the risk of double counts. In total, 95 bird species were recorded. However, here we only considered species breeding locally, which are more likely to be affected by local environmental conditions. After eliminating rare species (representing less than 1% of the total counts), non-breeding species (migrants), raptors (which have large territories) and gregarious feeding species that breed in towns and villages but feed in farmland landscapes (e.g., common swift *Apus apus*; see S1Appendix. for detailed species selection), 40 species were left for the rest of the analysis.

### Landscape characterization

The land use of the study area was surveyed twice a year from 2009 to 2013 corresponding to the periods for early harvesting and late sowing of crops. All data were geo-referenced and mapped into a GIS geodatabase. Spatial data were processed using Quantum GIS version 1.7.3 (Development Team 2002–2010). We identified 37 land use types based on the field survey (33 agricultural, 3 urban and 1 forest). These land uses were regrouped into 11 categories: alfalfa, grassland, ryegrass, sunflower, spring crops of pea-flax-field beans, rapeseed, cereals, maize, other crops (e.g. mustard, sorghum, millet and tobacco, representing less than 2% of crops), urban, and forest. This clustering was based on expert opinion to allow simplifying the analysis and obtaining a more functional classification according to species preferences. Two linear components (roads and hedgerows) were added to these categories, as we suspected that these played an important role structuring bird communities in an agricultural landscape. For each point count station, we recorded the area occupied by each land use category as well as the length of hedgerows and roads or paths within a buffer area around it. A previous study [31] found that the best scale for assessing the environmental effect on farmland birds at this study site, during the breeding season, was an average of 300 meters. However, this scale varied slightly between species; we therefore choose to assess the effect of environmental variables at various radii ranging from 200m to 1400m in 200m intervals (resulting in 7 grains of analysis).

### Statistical analyses

#### Step 1: Farmland bird metacommunity patterns

The distribution patterns of species within the metacommunity were first analyzed using the methods described by Leibold and Mikkelson and Presley et al. [5,27]. This method characterizes metacommunities using a site-by-species matrix, to identify particular idealized metacommunity patterns that can be related to the underlying assembly mechanisms (biotic interactions, environment or random assembly). Note, however, that this method cannot assess the potential role of dispersal, which is central to metacommunity dynamics [19].

**Fig 2:**
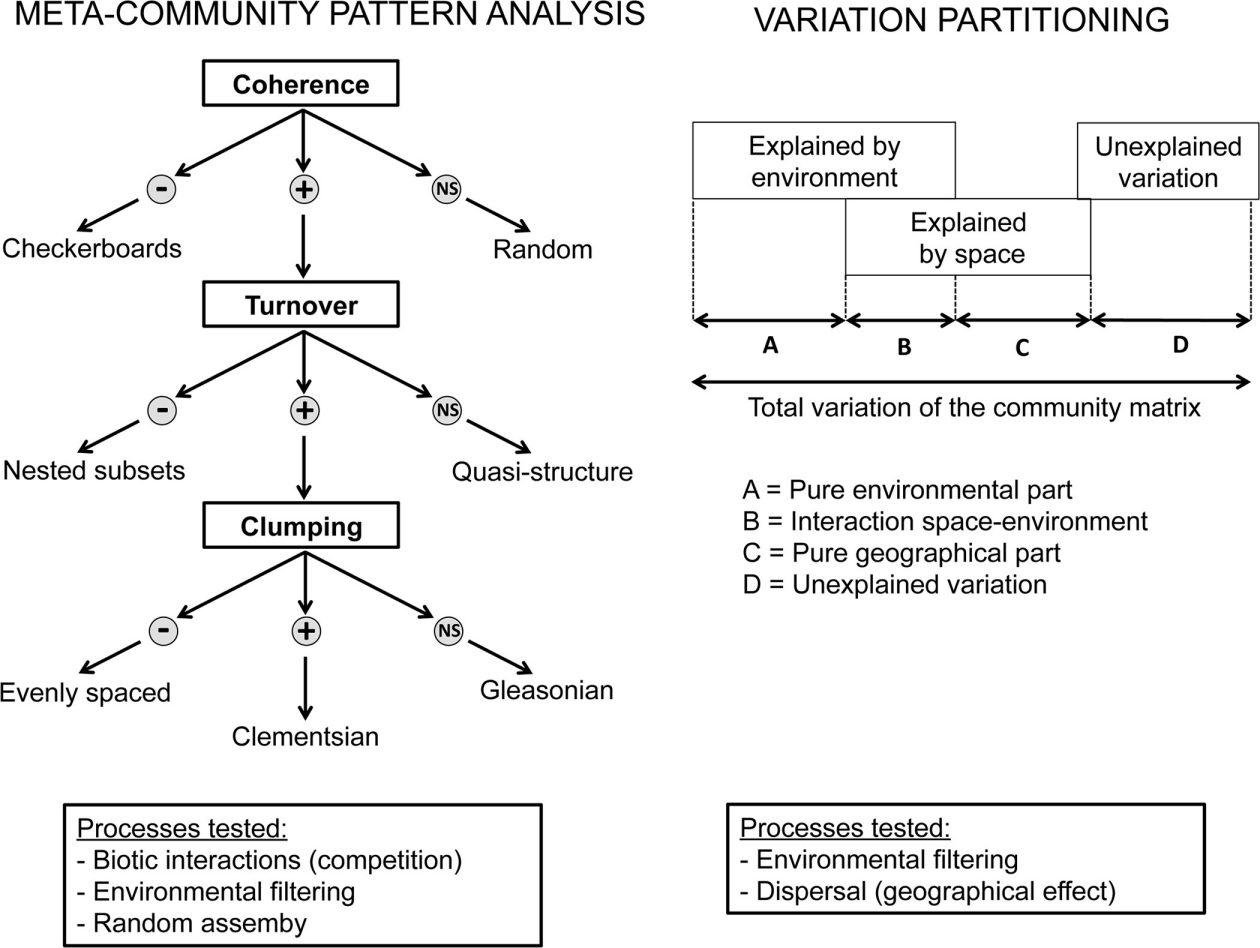
Description of methodology. Principles and comparison of the two methods: metacommunity pattern analysis characterizes the patterns generated by environmental factors, biotic interactions or random processes whereas variation partitioning can distinguish the effect of environment factors and dispersal on the community assembly.

First, we ordered the site-by-species matrix according to the first or second axes obtained after a correspondence analysis (using a reciprocal averaging algorithm). This analysis maximizes the proximity of sites with similar species composition and the proximity of species sharing the same sites. The reordered matrix thus maximizes the coherence of the species distribution prior to assessing the deviation from a null model [5]. Then, the ordered matrix was used to calculate three indices to characterize the metacommunity structure: coherence, species range turnover, and boundary clumping (Fig 2), (see [5] for a full description of the method to compute these indices). First, coherence indicates whether the various species are non-randomly distributed along a particular gradient (positive coherence), are mutually excluded owing, for example, to interspecific competition (negative coherence, with a characteristic checkerboard pattern) or are randomly assembled (coherence close to zero, i.e., non-significant). When coherence is positive, two further indices need to be calculated. The second index corresponds to the spatial species turnover, which measures the number of times one species replaces another one between two sites. The number of replacements is calculated for each pair of sites and each pair of species. A negative turnover, characterized by less replacement than expected to occur by chance, suggests a nested structure. If the turnover is positive, a third index is calculated: the boundary clumping. This indicates the aggregation of the distribution ranges between species. When positive, the pattern is Clementsian, i.e., different groups of species share the same ecological boundaries (indicating species that have the same environmental tolerances). When not significant, the pattern is Gleasonian, i.e., each species has its own ecological boundaries so the species differ in their environmental tolerances. A negative value indicates an evenly spaced distribution, showing that there are significant differences in environmental tolerances between species. These three indices (coherence, spatial species turnover and boundary clumping) were then compared against the values expected for purely random variations to assess their significance (see details of the null model below).

All the analyses were calculated using the Matlab “metacommunity” function developed by Presley et al. [5] (available from http://faculty.tarleton.edu/higgins/metacommunity-structure.html). The function can be used to test several null models. Here we used a null model where the species richness per site was fixed (equal to the observed richness), but the probability that a particular species occurred at that site varied according to the occurrence of that species (i.e., the most common species having a higher probability of occurrence). This model assumes that sites differ in suitability, and takes into account the commonness of each species (proportional occurrence). We performed 200 iterations (default parameter), see [32,33] for details.

For this first analysis, the data from each point count station (10 counts at each station) were pooled. The mean abundance of each species at each point count station was used as a proxy for their local abundance. Because land use varied between years, both the mean and the coefficient of variation of % composition for each environmental variable over the five year study period were analyzed in order to account for both the mean composition and the temporal variability of the landscape. Although the ordination is based only on species occurrences, the axes of the correspondence analysis are assumed to be related to a latent environmental gradient [34]. The correspondence with a real environmental gradient can be verified subsequently, by checking the correlation with measured environmental variables. Therefore we calculated the Pearson correlation between the first two axes of the reciprocal averaging and the various environmental variables (mean and coefficient of variation between the 5 years), measured at each site (i.e., the environmental gradient) and at each spatial scale (S4 Appendix) as well as the correlation with the species richness gradient (gradient of species richness between the different local communities).

#### Step 2: Variation partitioning

Variation partitioning [35,36] was used to decompose the total variation of community composition into the parts explained purely by environmental, spatial or temporal predictors as well as their interaction (Fig 2). This method allowed assessing the pure spatial structure that was not explained by environmental variables, the latter being attributed to spatial activities of individuals, such as dispersal [7,13,37]. Variation partitioning was based on multiple partial redundancy analyses (RDA), including environmental, geographical or temporal variables only or in combination. The fractions of the partitioning are then obtained by simple subtraction [37].

RDA is a constrained ordination method that can be used to analyze the community composition matrix with respect to explanatory matrices. It can be seen as an extension of multiple linear regressions for multivariate data. Abundance data were, first, Hellinger transformed. This transformation is recommended to reduce the asymmetry of community data containing many zeros prior to an ordination method such as RDA [35,38]. Geographical axes were obtained by Trend Surface Analysis (TSA) using a third degree polynomial of the geographic coordinates of the point counts (x and y coordinates), and nine different spatial functions were tested (x^3^, y^3^, xy^2^, yx^2^, x^2^, y^2^, xy, x, y; see [39]). This made it possible to model the spatial structure at different spatial scales by testing both linear and non-linear functions, but without considering excessively complex variations. Indeed, if too many functions are tested, there is a risk of interpreting random variations as a spatial effect, artificially increasing the spatial contribution; see [40]. In a preliminary analysis, we also compared this method with Principal Coordinates of Neighbour Matrices (PCNM), which revealed very similar qualitative results (S5 Appendix).

Both year and session were included as temporal predictors. Environmental and spatial variables were selected independently for each scale using a forward selection procedure to reduce the number of variables while keeping the variation explained by these variables to a maximum. This analysis used the “forward.sel” function of “packfor” R package (R.3.1.0, 2014) (available from R-forge: http://r-forge.r-project.org/R/?group_id=195), and the “varpart” function of the “vegan” package for the variation partitioning. This analysis was carried out for each environmental scale from 200 to 1400, in 200m intervals.

#### Step 3: Breaking down diversity patterns into landscape and species classes

In the above stages, the dataset was analyzed as if it were homogeneous. However, there were considerable differences in both landscapes and bird communities in the study area. For instance, landscapes ranged from highly intensive open fields without any trees, to mosaics of plots delimited by hedges. In the same way, the data set includes many species representing a wide panel of habitat preferences. To refine our findings, we explored whether results remained valid with habitat and species classes based upon major shared attributes. Environmental and spatial variables were selected independently for each habitat or species group and each spatial scales.

Species classes were based on co-occurrence patterns in the data set. To build the classes, the “dudi.coa” function of the “ade4” package (R.3.1.0, 2014) was used to perform a correspondence analysis (as described above). The species distribution (mean and standard deviation) was then ordinated along the first axis of the correspondence analysis which explained most of the variation (17.65%, see S1 Appendix), using the function “sco.distri” package (ade4, see S2 Appendix). The ordination values for the species along the first ordination axis were then transformed to Euclidean distances, and a dendrogram (hierarchical clustering) was built using these distances (“hclust” function, “stats” package, R.3.1.0, 2014; S2 Appendix). The dendrogram was used on the 40 bird species to define the three *a posteriori* classes. This partitioning aims to refine the analysis by controlling the main structuring gradient. This ordination axis could be interpreted as corresponding to a tree cover gradient, as we observed strong correlation with a wooded gradient (see result), so the first class was qualified as “open-land species” (7 species, N = 2327 observations), the second as “intermediate” (17 species, N = 1919 observations) and the third as “woodland species” (17 species, N = 1438 observations).

Due to the strong observed effect of the tree gradient on bird distributions, and because hedgerows are well known to shape bird communities in open landscapes, e.g. [41–43], the analyses were also refined by repeating each analysis for three classes of landscapes separately. The three classes of landscapes were defined from the density of tree cover. Hedgerows were assumed to have a similar effect to small patches of trees for farmland birds, so the two variables were combined (the linear shapefile of hedgerows was converted to polygon by allocating a width of 100m along all hedgerows). This was chosen based on tree avoidance distance for open-land species such as skylark *Alauda arvensis* [31], and to give more weight to linear components when pulling with surface areas. Finally, the area of tree cover (hedgerows and forest) around each point count station was calculated in a 200 m buffer zone around point counts. The three classes corresponded approximately to 0–35% (open landscapes, N = 756 observations), 35–70% (intermediate landscapes, N = 766 observations), 70–100% (wooded landscapes, N = 1068 observations) of tree cover, and had similar sample sizes (i.e. 76, 77 and 107 sites, respectively). Wooded landscapes comprised small fields, with a high proportion of perennial crops and a large number of hedges and forest fragments.

## Results

### Structuring processes at the metacommunity level

Our farmland bird community showed a clear Clementsian pattern along the first two axes of the reciprocal averaging (S3 Appendix): all species were distributed along the same environmental gradient (positive coherence), with a species replacement along this gradient (positive turnover), and apparent clusters of species sharing the same habitat preferences (positive boundary clumping). The environmental gradient structuring the reciprocal averaging axis corresponded to area of tree cover (Pearson correlation with the hedgerow/forest gradient, r = 0.87, p<0.0001, with hedgerow only r = 0.74, p<0.0001 and with forest only r = 0.32, p<0.0001), see S2 Appendix and S4 Appendix. Species richness at each point count station was also highly correlated (r = 0.83, p<0.0001) with this environmental gradient, indicating that more species were found in wooded landscapes (S2 Appendix). Bird communities also showed a Clementsian pattern along the second axis of the reciprocal averaging, which was correlated with urban land use (r = 0.49, p<0.0001) see S3 Appendix and S4 Appendix.

**Fig 3:**
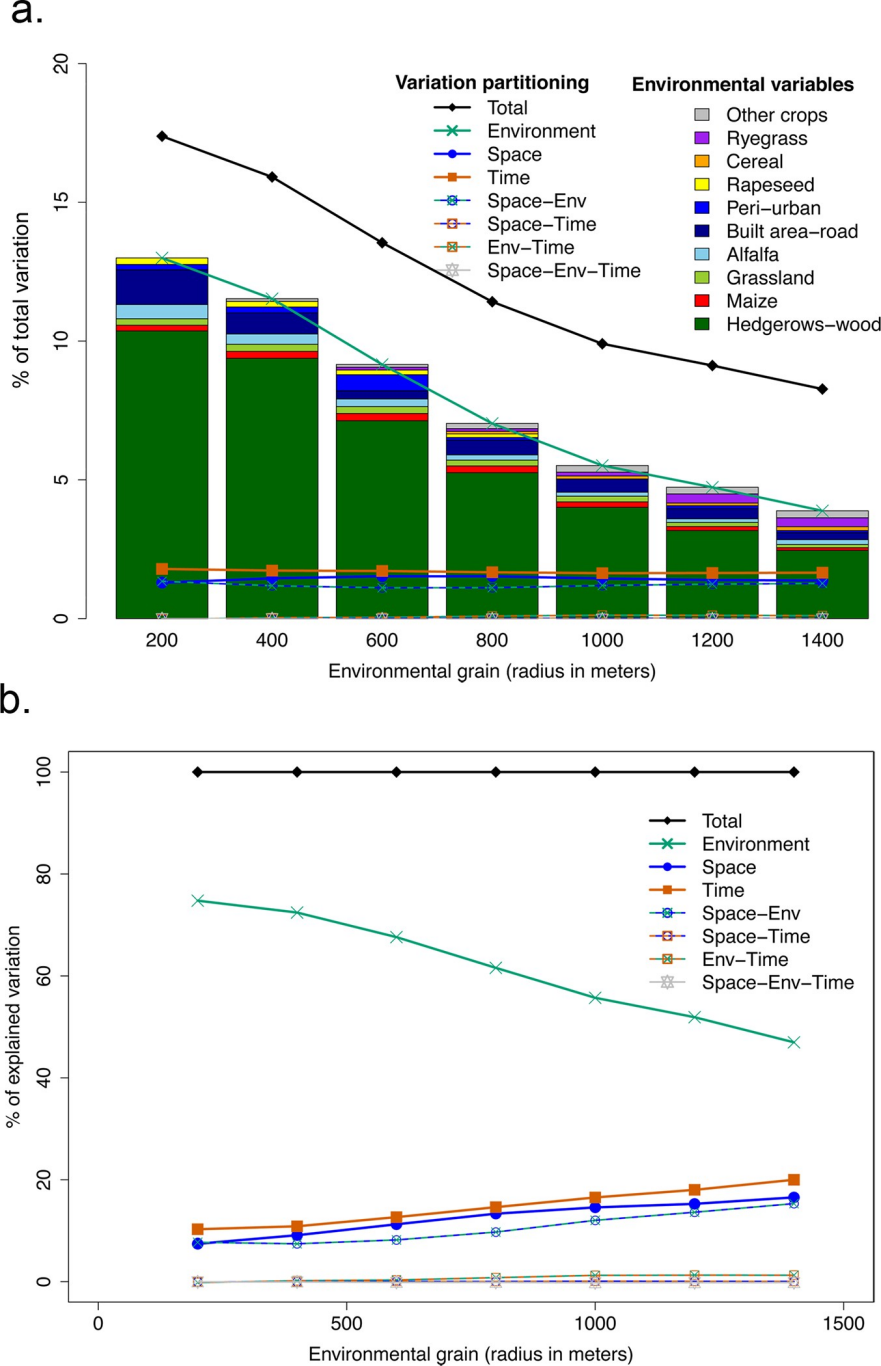
**Global variation partitioning analysis (all species classes and all landscape classes)**. **a. In percentage of the total variation b. In percentage of the explained variation**. The variation explained by each variable (geographical = blue curve, environmental = green, temporal = red curve), and interaction are represented with respect to the total explained variation (black curve), at each environmental grain (x-axis). The barplot represents the relative effect of each environmental predictors based on the F-ratio.

Variation partitioning made it possible to assess the relative importance of spatial, temporal and environmental factors in explaining bird community assembly (Fig 3). The total explained variation reached 17.4% (at 200 m), but decreased to 8.3% with increasing spatial grain (black curve, Fig 3). The relative contribution of each environmental variable was assessed using their F-ratio and represented as barplots below the curve. The environmental contribution was mainly explained by the presence of hedgerows (dark green bars in Fig 3A): hedgerows and forests represented 80% of the variation explained by environment at a 200m scale, and 63.3% at the 1400 m scale (Fig 3). In proportion of the total explained variation, the pure environmental (green curve), pure temporal (red curve) and pure geographical (blue curve) respectively represented 74.8%, 10.3% and 7.4% (Fig 3B). Therefore, most of the explained variation was accounted for by environmental variables.

### Spatial versus habitat effects using species class deconstruction

Species were grouped according to their habitat use based on the initial ordination axis (all species confounded), and therefore there was no a priori hypothesis on species traits or life history. However, a posteriori check reveals that these groups are ecologically relevant: the ordination axis was highly correlated with a tree cover gradient, so we defined these group as “open-land species”, “intermediate” and “woodland species”. Species within each group shared a combination of specific traits, see S7 Appendix. The open-land group is composed of ground nesting and migratory birds, with large body size for half of them, and all insectivorous species (adults or chicks, or both). By opposition group 3 (“woodland”) contains species with smaller body size, mostly nesting on trees or cavities, and mostly sedentary. As expected, when the bird community was split into these three classes, the Clementsian pattern disappeared, with each class showing a random structure along the first axis (S3 Appendix), though a Clementsian structure was still observed along the second axis for intermediate and woodland species.

**Fig 4:**
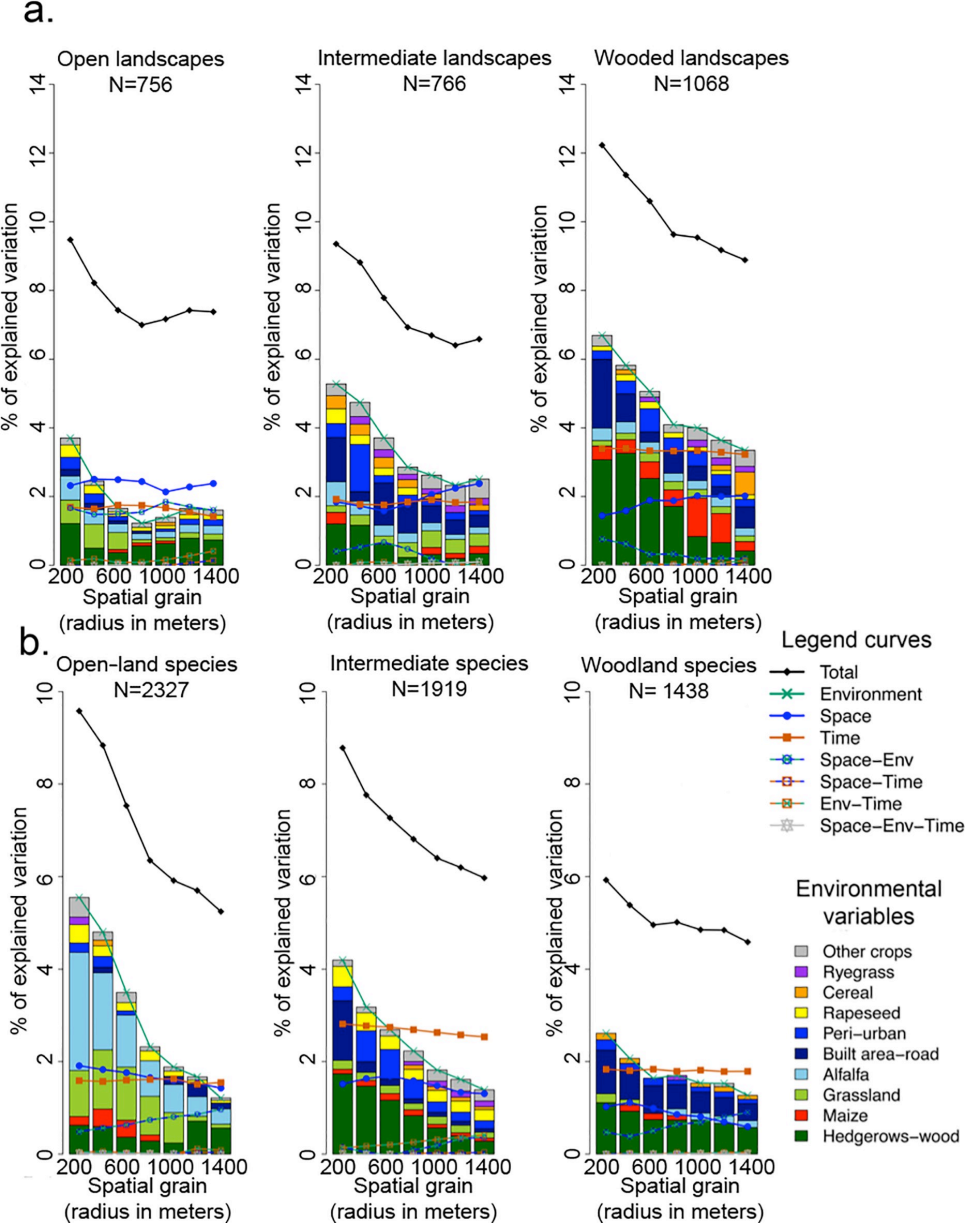
Variation partitioning per landscape and species class (% of explained variation). The curves represent the part of the explained variation by each variable: total (black), environmental (green), geographical (blue), temporal (red) and all interactions, at each environmental grain (x-axis). The barplot represents the relative effect of each of the environmental variables (based on the F-ratio). A. Partitioning per landscape B. Partitioning per species.

When partitioning per species, the models now only explain between 6% (wooded landscapes) and 9.5% (open landscapes) of the variance at 200m. The explanatory power then decreases when increasing grain size. While the environmental part still composes most of the explained variation for open-land species, the role of environment decreases from open-land to woodland species (S6 Appendix. and Fig 4B), explaining 5.5% of the variation for open-land species to only 2.6% for woodland species. Open land species respond more strongly to crop composition (especially grassland/alfalfa and rapeseed) than intermediate or woodland species, especially at smaller spatial grains (Fig 4B). Temporal variation appears constant across grain size and for each species group, while being proportionally more important for intermediate and woodland species, but remain overall quite low (around 2%). But if the deconstruction approach allows highlighting new structuring factors not visible in the global analysis (eg. crop composition for open-land species), our results globally show that when clustering by relevant species group, most of the variation seems now unexplained (result confirmed by both the meta-community pattern analysis and variation partitioning).

### Spatial versus habitat effects using landscape deconstruction

When the same analyses were carried out separately for each class of landscape, the community structure still showed Clementsian patterns (see S3 Appendix) for open and wooded landscapes along the first axis, but was random for intermediate landscapes.

With this deconstruction, our models only allowed explaining from 6.5% (Intermediate landscapes at 1200m) to 12.2% (wooded landscape at 200m) of the variation, the spatial part representing around 2% of the total variation. Relative importance of environmental and geographical factors in explaining the variation in bird community also depended on landscape class (Fig 4A and S6 Appendix): in open landscapes (dominated by annual crops), the geographical component represented in average 2.3% of the total variation (so 30% of the explained variation) against 1.9% for the environment (24% of the explained variation), so the spatial component globally exceeded the environmental contribution except at the smallest spatial scale. In contrast, in wooded landscapes, the environmental factors were dominant for all spatial grains. In intermediate landscapes (mosaic of annual crops and hedgerows), the geographical factor was as important as environment only at large spatial grains.

The temporal factor remains relatively quite high in all landscapes, in comparison to other factors, but tends to slightly increase in absolute value from open landscapes to wooded landscapes. The deconstruction per landscape appears globally efficient to remove the dominant effect of wooded components, although this effect does not totally disappear, especially in the most wooded landscapes (wooded components still accounting for 45.9% of the variation explained by environmental variables at 200 m in wooded landscapes), see Fig 4A. Landscape deconstruction, therefore, revealed the effect of additional environmental factors that did not appear in the global metacommunity analysis, such as the importance of alfalfa/grassland and rapeseed in open areas, and the proximity of urban area, alfalfa/grassland and other crops in wooded landscapes.

## Discussion

Overall, our analyses show that the stochasticity have a prominent place in our system. Indeed, although the accuracy of environmental predictors and the deconstruction approach, a large amount of variation in community composition remains unexplained throughout all analyses, with as little as 6.5% and a maximum of 20% being explained by environment, spatial structure, seasonal or annual variation or by the interaction of these factors. This unexplained variation can be due either to nondeterministic fluctuations, to unmeasured variables (biotic or abiotic) or to spatial structures that are too complex to be described by our geographical functions [39].

The extent of our study area may also influence these results. Other studies have found that increasing the scale of analysis may change the total variance explained in community composition and / or diversity [19], the percent explained usually increasing at larger spatial scales. Here we varied the grain of the environmental predictors but, because of the nature of the study area, could not have varied too much the extent. But we still observed a decrease of explained variation from global analysis to the deconstruction per landscape type. The fact that the variance explained decreases when sites are grouped by landscape type confirms that landscape type was indeed a meaningful and powerful predictor in the first place. However, exploring what happens under the deconstruction approach allows investigating further whether or not there are other new predictors that appear as important once landscape type has been controlled for.

Among the deterministic processes involved, both variation partitioning and community pattern analysis showed that environment, especially as related to tree cover (hedgerows and forests), represent the main part of the explained variation of bird communities in agricultural landscapes and masked other variables at global scale. A dominant effect of environmental variables on bird community composition has been shown in numerous other studies at different spatial scales [20,21,44–46]. Here we also found a stronger effect of environmental variables at fine spatial grain (200m), a scale that matches better the bird territories in the breeding period, which usually range from 100 to 1000 m radius [47,48]. By contrast, the effect of the spatial component remained low and relatively constant at all spatial scales. The effect of tree cover on farmland birds has been clearly demonstrated [41,49], as trees and hedgerows provide key nesting habitats and song-posts for many bird species [50], while they may be avoided by other species, e.g., skylarks and yellow wagtails [31,51,52]. So the dominant effect of environmental variables and the low spatial component observed suggest that dispersal limitation is not a main driver of community assembly at global scale whatever the grain size, but that local habitat heterogeneity is key, especially as related to the existence of wooded areas.

Using the deconstruction approach, both variation partitioning and metacommunity pattern analysis leave a large proportion of variance unexplained, even more so than using a global analysis approach. This is probably due to the fact that our environmental correlates exhibit less variability when the landscape is deconstructed into categories, whereas our biological response variable does not. The dominant effect of environment at global scale and the increase of stochasiticity at local scale have also been shown for bird communities in another study on a coastal area, see [53]. In the deconstruction per species group, here again the ordination appear quite efficient to define species groups sharing similar habitat requirements. Meta-community pattern analysis seems a good method to reveal the dominant large scale ecological processes involved, and can constitute a useful step to allow a deconstruction at finer scales.

Indeed, the deconstruction approach revealed additional processes, both resulting from landscape types or species groups, a likely consequence of a strong species turnover of the community along forested gradients. The deconstruction per species show that woodland species respond more strongly to environmental predictors than open-land species. Despite of the deconstruction, the abundance of forest fragments and hedgerows remain the main predictor for woodland species. The quantity and quality of these semi-natural habitats may be so also an important criteria. By opposition the importance of crop composition appear now more clearly for open-land species.

But beyond only a preference in habitat type, these three groups tend also to associate species sharing specific traits. Many other studies have also highlighted the relevance of using species traits to explain community assembly patterns [54,55]. Most of the existing literature concern aquatic organisms and tend to show that ecological determinism, and especially the relative influence of environment compared to dispersal, increases with dispersal ability and body size of the organisms under study [56–59]. This could be explained by the fact that larger organisms are less plastic in their fundamental niche, or because more mobile species are able to track suitable environmental conditions better [57]. Our study suggests an opposite pattern (S7 Appendix), with a higher relative importance of spatial drivers for open-land species, a group that comprise only migratory or partially migratory species with, on average, larger body sizes, and comprising exclusively ground nesting species. The latter could make them more likely to disperse as compared to other groups (nesting in trees) since they need to cope with crop rotation. Therefore, this apparent contradiction with previous findings may just be explained by the fact that birds in our system are not restricted by dispersal limitation in itself (no limiting trait or physical barrier in environment) but are only constrained by tracking habitat changes.

The deconstruction per landscape type also reveal other processes. In wooded landscapes, environmental factors remained the main deterministic processes, whatever the spatial scale considered; in contrast in open landscapes, the spatial component exceeds the importance of environmental factors (except at the lowest spatial grain), explaining in average 2.3% of the total variation against 1.9% for the environment. Although we cannot totally exclude the possibility that the spatial effect results partly from the omission of some structuring environmental factors (such as agricultural practices) [60], this is unlikely to fully explain the observed patterns in our analyses. Indeed, the environmental data available here were highly detailed, and included land use for each single field, accurate mapping of hedgerows, wooded fragments and other semi-natural components, and changes through time for every year and land use type. Furthermore, the meta-community pattern analysis does not suggest a strong competition between species in our system (which would be characterized by a checkerboard pattern).

This pure spatial component that cannot be produced by the structuration of environmental variables may be attributed to spatial activities of individuals, such as adult dispersal and/or foraging activities [7,13,37]. Considering the high dispersal ability of birds, pure spatial structuration is expected to be low at local scale, as observed in other studies (eg. [20,53]). Here we suggest that this spatial effect may be explained by individual movements during the breeding season rather than by dispersal limitation *sensu stricto*. Theoretical and experimental studies suggest that dispersal is one of the possible strategies that can be selected in ecosystems characterized by a high spatiotemporal random environmental variation [61–63], a pattern expected with the crop rotations. Therefore, birds may need larger foraging habitats in less suitable areas, such as those represented by open habitats in agricultural fields. In addition, it has been shown that due to the low food availability in intensive farming areas, birds often travel further for foraging [64,65]. Supporting this idea, some long-term studies have indicated a dramatic decline of insects in Europe over the last decades [66,67], pattern that seems to be at least partly linked with farmland intensification.

If the high unexplained variation partly results from the loss of environmental heterogeneity with the partitioning per habitat, we still observed different patterns between wooded and open landscapes. In our study, the pattern observed in open landscapes suggests that bird community assembly in highly disturbed agricultural landscapes is more largely determined by stochasticity and spatial effects than in more perennial landscape. Similar conclusions seems to apply in urban areas [68]. In such highly unpredictable and changing landscapes, dispersal seems more important to deal with uncertainty, but can be not sufficient and result in ecological traps as suggested by some previous studies [69,70]. But, more generally, high stochasticity seems more the rule than the exception, a phenomena that also applies to more natural ecosystems [54,55]. This high unexplained variation may also partly result of imperfect detection. Although it has been shown in previous studies that 5 minutes point counts are usually sufficient to provide good estimates of species occurrences and community composition in open habitat like farmland, we are still unlikely to detect all individuals [29,30].

## Conclusion

Our results highlight the critical need of conducting multi-scale studies and to consider several processes acting on the metacommunity at the same time. Using a combination of different methods and adopting a deconstruction approach can help improve our understanding of this complex set of community drivers.

Put in context, this study supports the idea that stochasticity and historicity are probably still very important components at the landscape scale. Part of the observed patterns are likely driven by unexpected fluctuations in population dynamics and space occupancy, while other sources of explanation might be found in the history of how individual bird territories came to be, such as colonization and extinction history, as well as lagged responses to major environmental changes [71].

Our results also emphasize the importance of wooded areas and landscape structure to explain community assembly of farmland birds. But they also highlight a higher role of dispersal in open habitats, which are more highly disturbed and unpredictable, and further suggest the importance of preserving source areas of biodiversity and maintaining landscape connectivity in these agricultural mosaics. Moreover, since open land species appear particularly sensitive to crop composition, and since there is a higher risk of potential mismatch between habitat preferences and breeding success in unpredictable landscapes, one may expect a positive effect of crop heterogeneity. Indeed a diversified landscape will more likely provide a suitable habitat and stable food resource at the territory scale [72,73]. However, our study only provided circumstantial evidence for this hypothesis, since results were based on spatial patterns. More direct tests of this hypothesis could be carried out by directly tracking animal movement in agricultural mosaics, and by further replicating spatially and temporally these analyses in other regions.

## Supporting information

S1 Appendix Species list (per class) and results of the redundancy analysis (RDA). We observe that the three species classes are ordinated along the first axis of the RDA, which is strongly correlated with a gradient of wooded component (see also S4 Appendix). This first axis explains 17.65% of the total variation, while the second axis explains 9.23%.
(DOCX)

S2 Appendix Analysis of species co-occurrence by correspondence analysis. Species are distributed along a tree cover gradient (strong correlation with the first axis of the correspondence analysis). A dendrogram can be built by transforming the ordination value along this axis into the Euclidean distance. Three species classes can be distinguished using the dendrogram, corresponding to openland, intermediate and woodland species.
(DOCX)

S3 Appendix Results of the meta-community pattern analysis on the first two axis of the Redundancy Analysis (RDA). (see Presley, Higgins and Willig, 2010 and Leibold and Mikkelson, 2002)
The table present the value of the different indices, computed with the « meta-community function » in Matlab (see Leibold and Mikkelson, 2002):
Abs = the number of embedded absences in a given ordinated matrix
Apr = pvalue associated with embedded absences
MA = mean number of embedded absences base on null models
SA = standard deviation of number of embedded absences based on null models
Re = number of replacements (checkerboard)
Rpr = pvalue associated with replacements
MR = mean number of replacements base on null models
SR = standard deviation of number of replacements based on null models
M = Morisita Community index value
Mpr = pvalue associated with Morisita index
The resulting pattern is indicated for each analysis (global analysis, by landscape class, by species class or with both partitioning), for the first two axis of the redundancy analysis.
(DOCX)

S4 Appendix Correlation between the RDA axis (first two axis) and the environmental variables at each environmental grain. (DOCX)

S5 Appendix Variation partitioning: comparison between two spatial models: Principal Coordinates of Neighbor Matrices (PCNM) and Trend Surface Analysis (TSA). Results are presented without the temporal component. In this analysis, all sessions and years have been pooled together. Because land use varied between years, both the mean (M) and the coefficient of variation (CV) of % composition for each environmental variable over the five year study period were analyzed in order to take account both the mean composition and the temporal variability of the landscape.
Although we observe similar patterns, PCNM give more importance to the geographical component and less to the environmental part.
(DOCX)

S6 Appendix **Variation partitioning for each class of landscapes (open, intermediate and wooded) (4a) and each class of species (open-land, intermediate and woodland species) (4b).** The curves represent the variation explained by each variable: environmental (green), geographical (blue), temporal (red) and all interactions in relation to the part of explained variation (black), at each environmental grain (x-axis). This figure aims to compare the relative contribution of the deterministic processes, keeping the part of explained variation constant (100%). Unexplained variation does not appear on this figure.
A. Partitioning per landscape
The relative contribution of the spatial component decreases from open to wooded landscapes: the geographical part has a stronger effect than the environmental part in open landscapes whereas the environmental part dominates in wooded landscapes.
B. Partitioning per species
Spatial factors appear stronger in proportion for open-land species in comparison to woodland species, but environmental effects remain dominant for each species classes.
(DOCX)

S7 Appendix Table of species traits. The groups correspond to those defined by the correspondence analysis (see partitioning per species). The values of the traits correspond to a mean value per species, defined according to the literature. *Sources*:
*1. Guide ornitho*, *Lars Svensson*, *Peter J*. *Grant*, *Killian Mullarney*, *Dan Zetterström*, *edition 2011*
*2. Website*: *Oiseaux*.*net*.
(DOCX)

S8 Appendix Database. The database contains all the data from the point counts and GIS data. This includes the ID of the point (“IdPoint”), the year and the session (2 sessions per year from 2009 to 2013). The abundance of each species is reported on the table. The first column (“Gradient_HedgerowsForest200m”) corresponds to the gradient of wooded components used to do the partitioning per landscape. This variable was computed by summing the area of hedgerows (considering a buffer of 100m around this linear component) and woodland. The data base also included the areas of the 11 categories of land use: alfalfa, grassland, ryegrass, sunflower, spring crops of pea-flax-field beans, rapeseed, cereals, maize, other crops (e.g. mustard, sorghum, millet and tobacco, representing less than 2% of crops), urban, and forest and two linear components (roads and hedgerows). For each point count station, we recorded the area occupied by each land use category as well as the length of hedgerows and roads or paths within a buffer area from 200m to 1400m. Finally, the database contains the 9 spatial functions (third degree polynomial of the geographic coordinates of the point counts) used in the variation partitioning analysis (“Spatial1” to “Spatial9”).
(XLS)
