# Supplementary figures and images for: On the relative importance of space and environment in farmland bird community assembly
Source: PLoS One. 2019 Mar 11;14(3):e0213360. doi: 10.1371/journal.pone.0213360 (PMC6411160; doi:10.1371/journal.pone.0213360)

**S1 Appendix
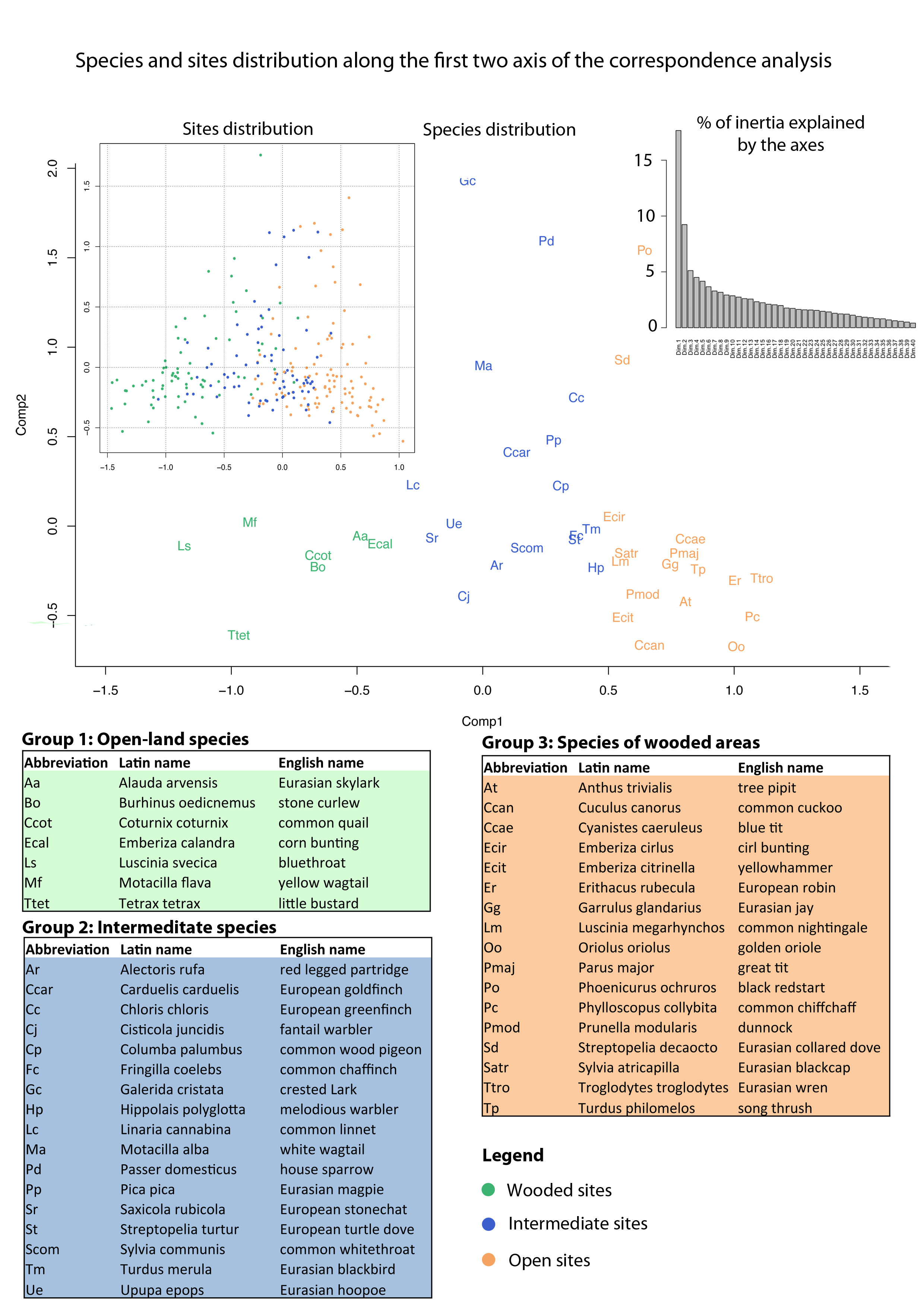
**

Supplement: S1 Appendix — We observe that the three species classes are ordinated along the first axis of the RDA, which is strongly correlated with a gradient of wooded component (see also S4 Appendix). This first axis explains 17.65% of the total variation, while the second axis explains 9.23%. (DOCX) [file pone.0213360.s001.docx]

**S2 Appendix**


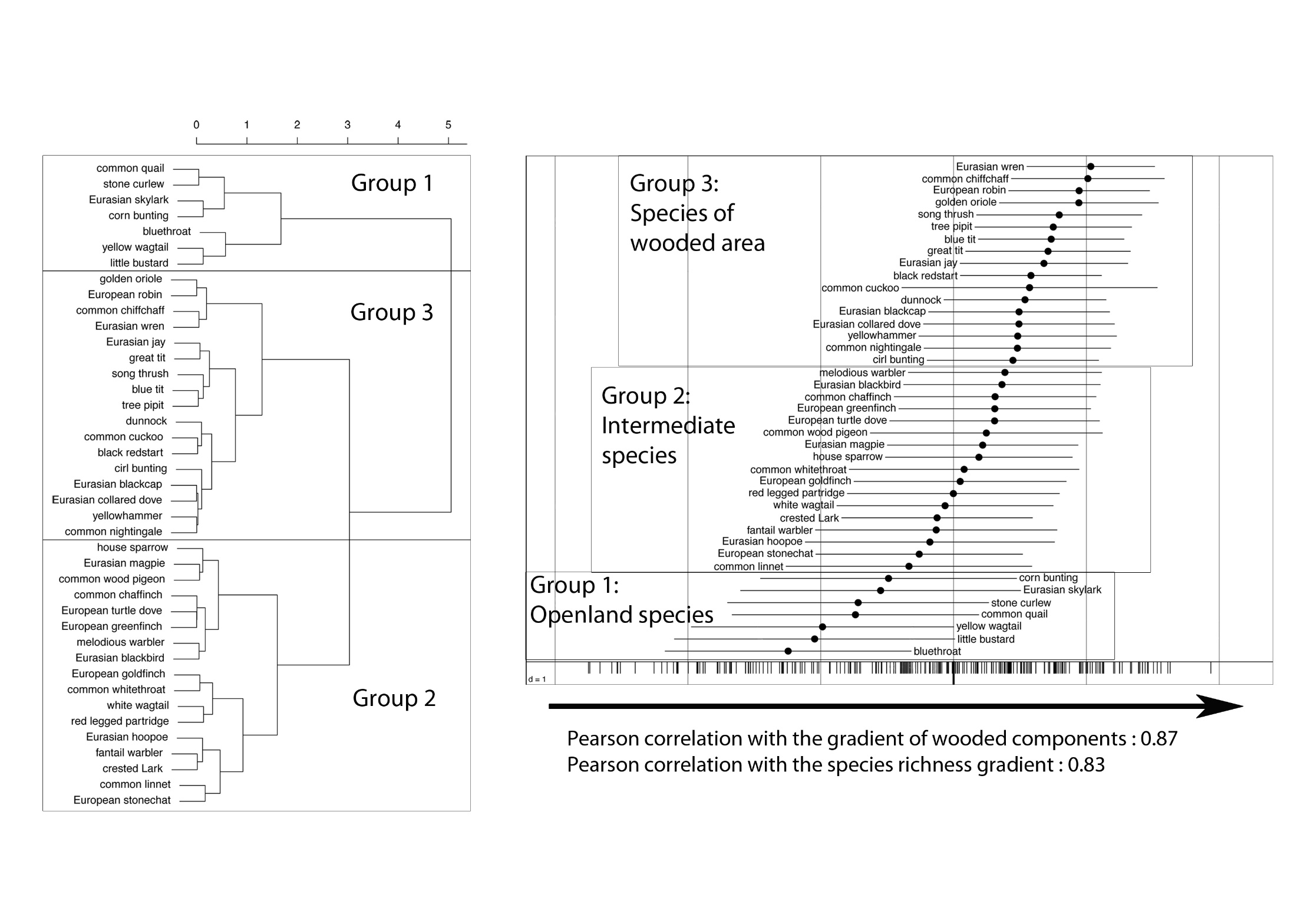

Supplement: S2 Appendix — Species are distributed along a tree cover gradient (strong correlation with the first axis of the correspondence analysis). A dendrogram can be built by transforming the ordination value along this axis into the Euclidean distance. Three species classes can be distinguished using the dendrogram, corresponding to openland, intermediate and woodland species. (DOCX) [file pone.0213360.s002.docx]

**S3 Appendix**


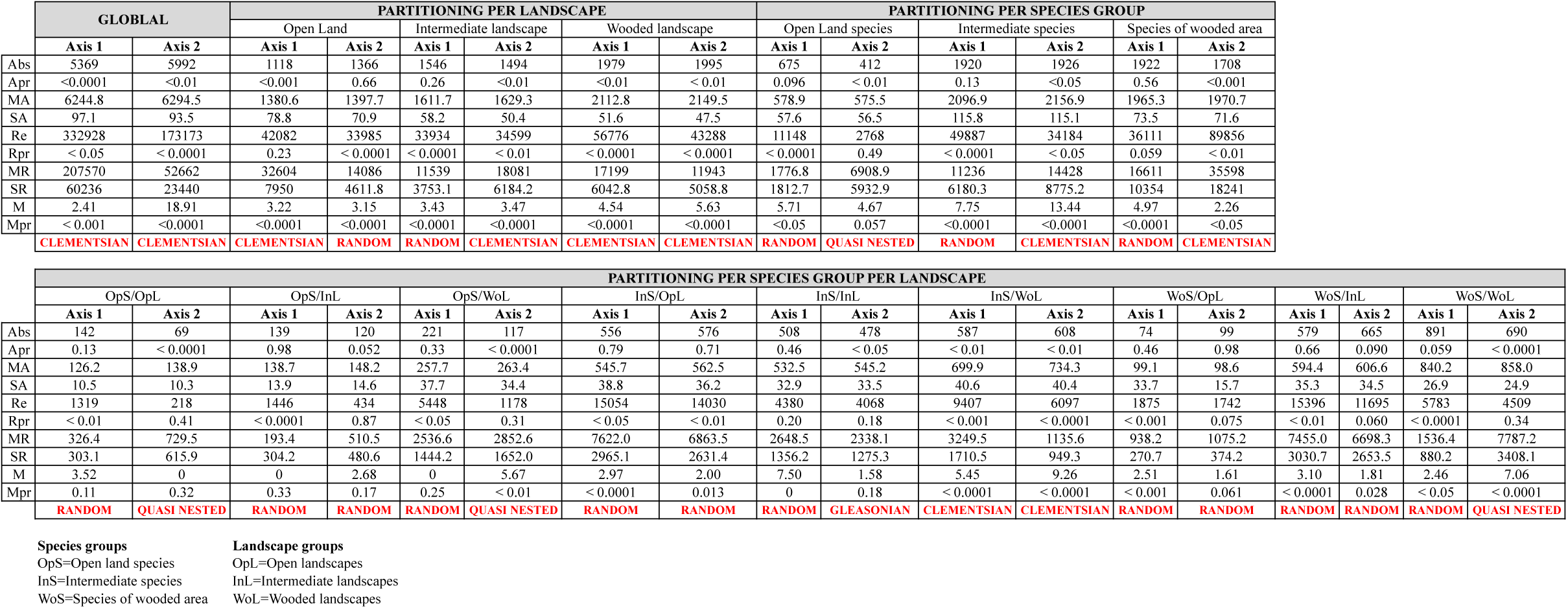

Supplement: S3 Appendix — (see Presley, Higgins and Willig, 2010 and Leibold and Mikkelson, 2002) The table present the value of the different indices, computed with the « meta-community function » in Matlab (see Leibold and Mikkelson, 2002): Abs = the number of embedded absences in a given ordinated matrix Apr = pvalue associated with embedded absences MA = mean number of embedded absences base on null models SA = standard deviation of number of embedded absences based on null models Re = number of replacements (checkerboard) Rpr = pvalue associated with replacements MR = mean number of replacements base on null models SR = standard deviation of number of replacements based on null models M = Morisita Community index value Mpr = pvalue associated with Morisita index The resulting pattern is indicated for each analysis (global analysis, by landscape class, by species class or with both partitioning), for the first two axis of the redundancy analysis. (DOCX) [file pone.0213360.s003.docx]

**S4 Appendix**


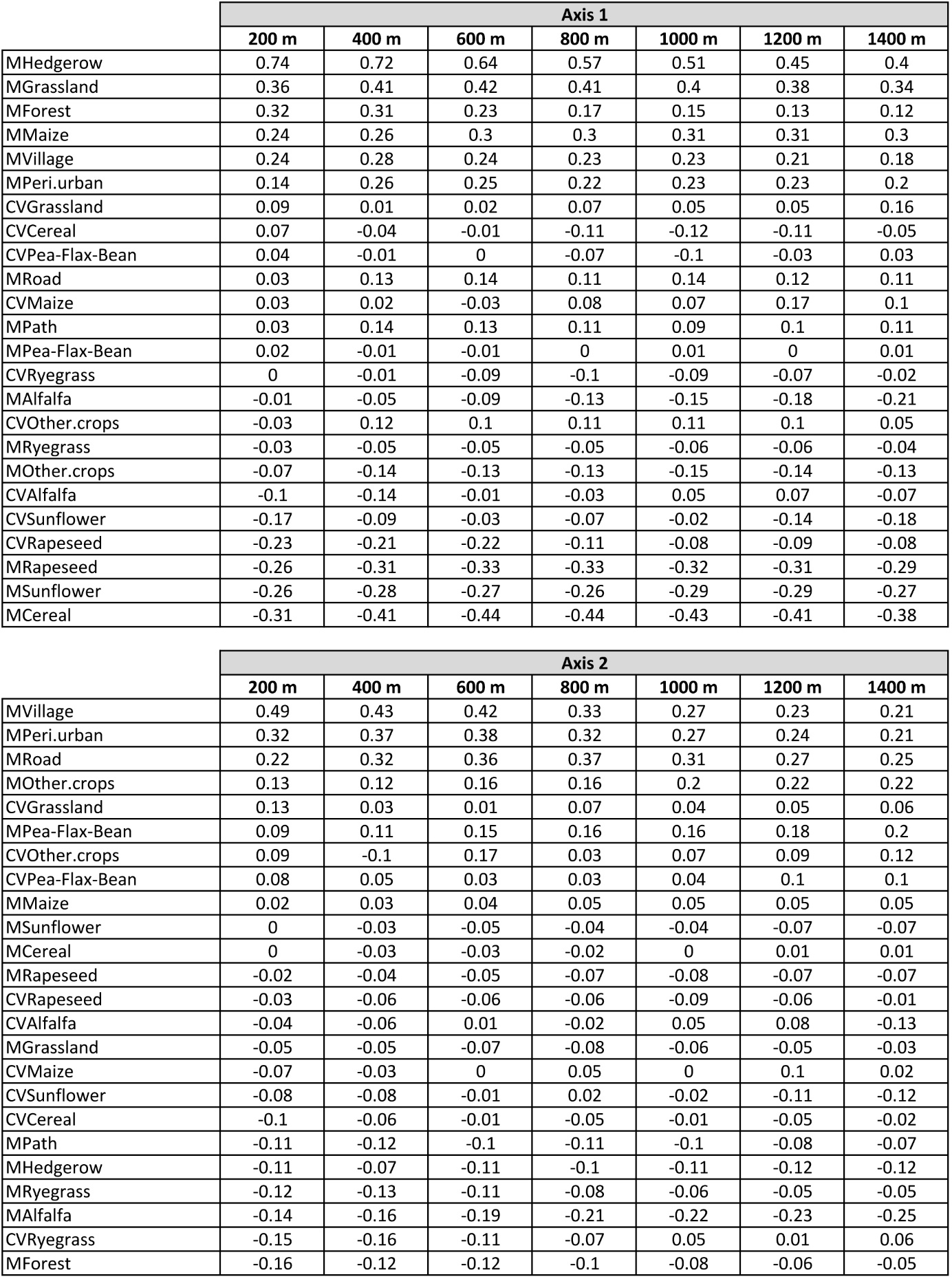

Supplement: S4 Appendix — (DOCX) [file pone.0213360.s004.docx]

**S6 Appendix**


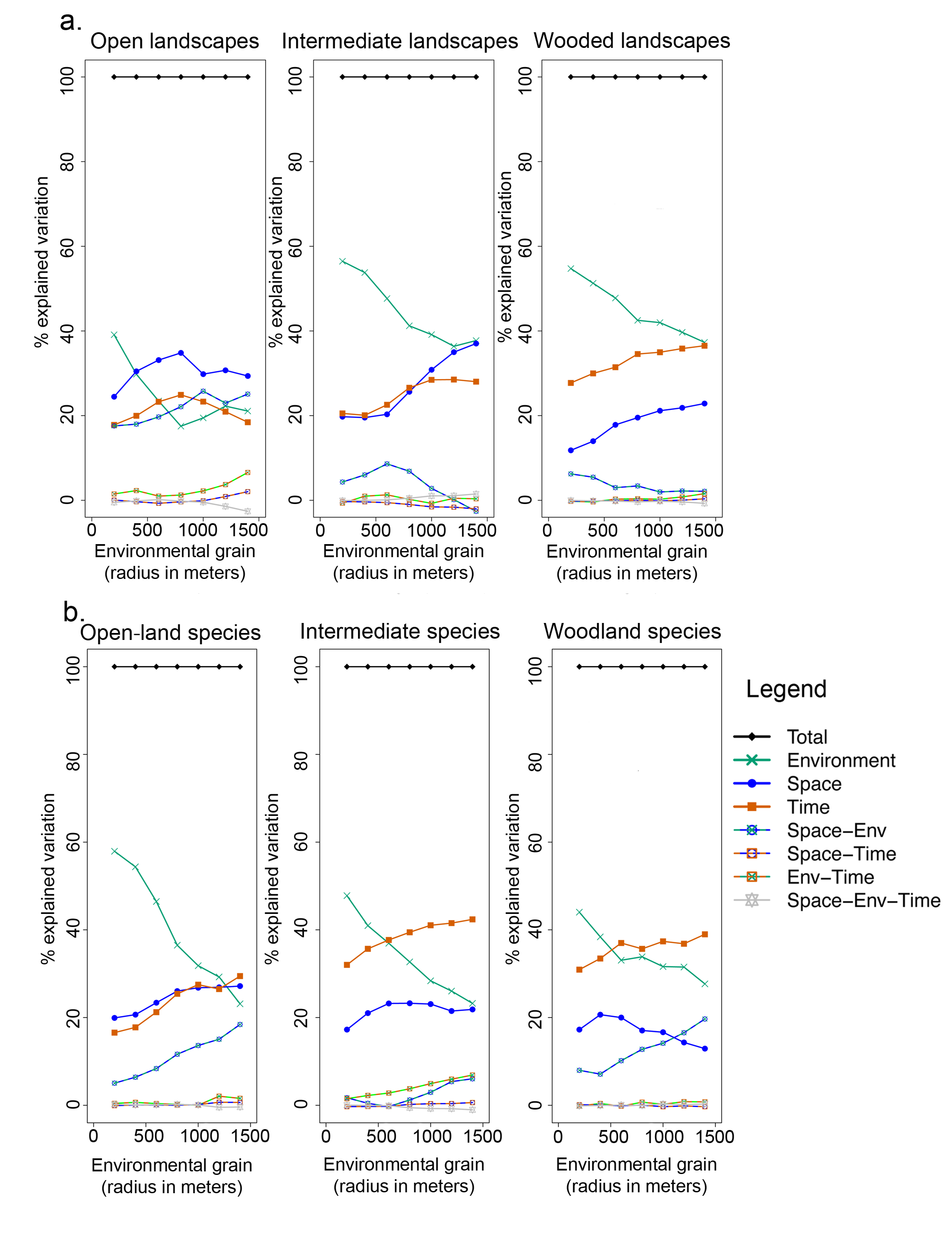

Supplement: S6 Appendix — Variation partitioning for each class of landscapes (open, intermediate and wooded) (4a) and each class of species (open-land, intermediate and woodland species) (4b). The curves represent the variation explained by each variable: environmental (green), geographical (blue), temporal (red) and all interactions in relation to the part of explained variation (black), at each environmental grain (x-axis). This figure aims to compare the relative contribution of the deterministic processes, keeping the part of explained variation constant (100%). Unexplained variation does not appear on this figure. A. Partitioning per landscape The relative contribution of the spatial component decreases from open to wooded landscapes: the geographical part has a stronger effect than the environmental part in open landscapes whereas the environmental part dominates in wooded landscapes. B. Partitioning per species Spatial factors appear stronger in proportion for open-land species in comparison to woodland species, but environmental effects remain dominant for each species classes. (DOCX) [file pone.0213360.s006.docx]
