# Supplementary material for: On the relative importance of space and environment in farmland bird community assembly
Source: PLoS One. 2019 Mar 11;14(3):e0213360. doi: 10.1371/journal.pone.0213360 (PMC6411160; doi:10.1371/journal.pone.0213360)

**S5 Appendix : Variation partitioning : comparison between two spatial models : Principal Coordinates of Neighbor Matrices (PCNM) and Trend Surface Analysis (TSA)**


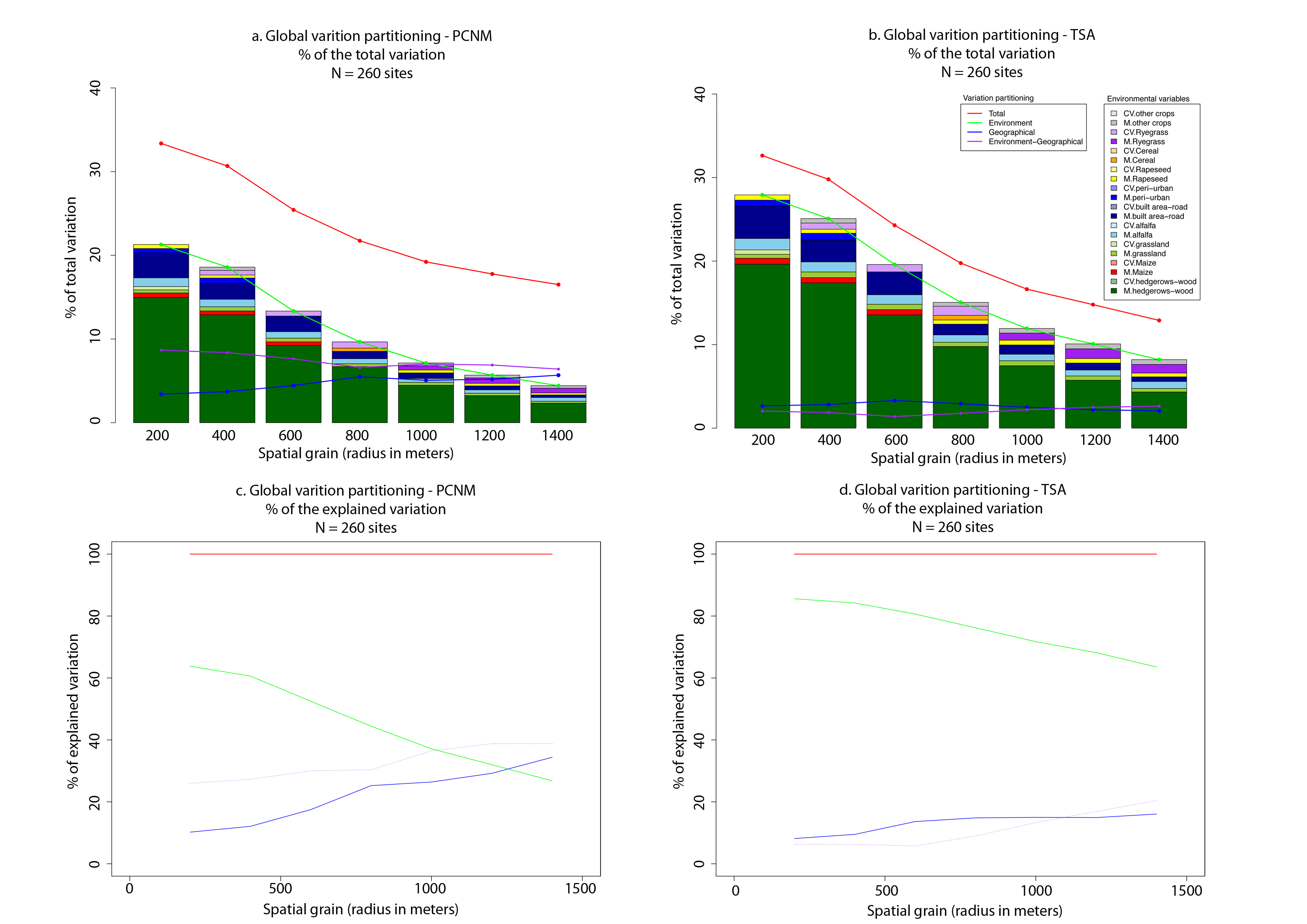

Supplement: S5 Appendix — Results are presented without the temporal component. In this analysis, all sessions and years have been pooled together. Because land use varied between years, both the mean (M) and the coefficient of variation (CV) of % composition for each environmental variable over the five year study period were analyzed in order to take account both the mean composition and the temporal variability of the landscape. Although we observe similar patterns, PCNM give more importance to the geographical component and less to the environmental part. (DOCX) [file pone.0213360.s005.docx]
