# Supplementary material for: On the relative importance of space and environment in farmland bird community assembly
Source: PLoS One. 2019 Mar 11;14(3):e0213360. doi: 10.1371/journal.pone.0213360 (PMC6411160; doi:10.1371/journal.pone.0213360)
Supplement: S7 Appendix — The groups correspond to those defined by the correspondence analysis (see partitioning per species). The values of the traits correspond to a mean value per species, defined according to the literature. Sources: 1. Guide ornitho, Lars Svensson, Peter J. Grant, Killian Mullarney, Dan Zetterström, edition 2011 2. Website: Oiseaux.net. (DOCX) [file pone.0213360.s007.docx]

**S7 Appendix: Table of species traits**

| **Group** | **Species** | **Ressources quantity** | | **Ressource type** | **Nesting place** | **Dispersion** |
| --- | --- | --- | --- | --- | --- | --- |
|  |  | **Body mass** | **Clutch size** |  |  | **Migration** |
| 1 | Alauda arvensis | 45 | 4 | insectivorous | Ground | Partial migratory |
| 1 | Coturnix coturnix | 110 | 10 | insectivorous | Ground | Migratory |
| 1 | Burhinus oedicemus | 410 | 2 | insectivorous | Ground | Migratory |
| 1 | Emberiza calandra | 46.5 | 4.5 | granivorous/insectivorous | Ground | Partial migratory |
| 1 | Luscinia svecica | 19 | 5.5 | insectivorous | Ground | Migratory |
| 1 | Motacilla flava | 23 | 5.5 | insectivorous | Ground | Migratory |
| 1 | Tetrax tetrax | 875 | 3.5 | insectivorous | Ground | Migratory |
|  | **Mean** | **218.4** | **5** |  |  |  |
| 2 | Passer domesticus | 30 | 5.5 | omnivorous | Cavity | Sedentary |
| 2 | Pica pica | 177.5 | 6.5 | omnivorous | Hedgerow-Tree | Sedentary |
| 2 | Columba palumbus | 485 | 2 | granivorous/insectivorous | Hedgerow-Tree-Ground | Migratory |
| 2 | Fringilla coelebs | 21.5 | 5 | granivorous/insectivorous | Hedgerow-Tree | Migratory |
| 2 | Streptopelia turtur | 187.5 | 2 | granivorous | Hedgerow-Tree | Sedentary |
| 2 | Chloris chloris | 29.5 | 5 | granivorous | Hedgerow-Tree | Partial migratory |
| 2 | Hippolais polyglotta | 12.5 | 4 | insectivorous | Hedgerow-Tree | Migratory |
| 2 | Turdus merula | 95 | 4.5 | insectivorous | Hedgerow-Tree | Sedentary |
| 2 | Carduelis carduelis | 16 | 4.5 | granivorous/insectivorous | Hedgerow-Tree | Partial migratory |
| 2 | Sylvia communis | 15 | 4.5 | insectivorous | Hedgerow-Tree | Migratory |
| 2 | Motacilla alba | 23 | 5.5 | insectivorous | Cavity | Migratory |
| 2 | Alectoris rufa | 425 | 13.5 | granivorous | Ground | Sedentary |
| 2 | Upupa epops | 67.5 | 6.5 | insectivorous | Cavity | Migratory |
| 2 | Cisticola juncidis | 8.5 | 4.5 | granivorous/insectivorous | Ground | Sedentary |
| 2 | Galerida cristata | 40 | 4 | granivorous/insectivorous | Ground | Migratory |
| 2 | Linaria cannabina | 17.5 | 5 | granivorous/insectivorous | Hedgerow-Tree | Sedentary |
| 2 | Saxicola rubicola | 15.5 | 5.5 | insectivorous | Ground | Migratory |
|  | **Mean** | **98.03** | **5.18** |  |  |  |
| 3 | Oriolus oriolus | 71.5 | 3.5 | insectivorous | Hedgerow-Tree | Migratory |
| 3 | Erithacus rubecula | 19 | 6 | granivorous/insectivorous | Hedgerow-Tree | Sedentary |
| 3 | Phylloscopus collybita | 7.5 | 6.5 | insectivorous | Hedgerow-Tree | Migratory |
| 3 | Troglodytes troglodytes | 10.5 | 6 | insectivorous | Cavity | Sedentary |
| 3 | Garrulus glandarius | 165 | 5 | omnivorous | Hedgerow-Tree | Sedentary |
| 3 | Parus major | 18.5 | 9 | granivorous/insectivorous | Hedgerow-Tree | Sedentary |
| 3 | Turdus philomelos | 77.5 | 4.5 | granivorous/insectivorous | Hedgerow-Tree | Sedentary |
| 3 | Cyanistes caeruleus | 10.5 | 11 | granivorous/insectivorous | Cavity | Sedentary |
| 3 | Anthus trivialis | 22.5 | 5 | insectivorous | Ground | Migratory |
| 3 | Prunella modularis | 20 | 4.5 | granivorous/insectivorous | Hedgerow-Tree | Sedentary |
| 3 | Cuculus canorus | 117.5 | 9 | insectivorous | Hedgerow-Tree | Migratory |
| 3 | Phoenicurus ochruros | 17 | 5 | insectivorous | Cavity | Migratory |
| 3 | Emberiza cirlus | 21 | 3.5 | granivorous/insectivorous | Hedgerow-Tree | Sedentary |
| 3 | Sylvia atricapilla | 17 | 4.5 | granivorous/insectivorous | Hedgerow-Tree | Partial migratory |
| 3 | Streptopelia decaocto | 187.5 | 2 | granivorous/insectivorous | Hedgerow-Tree | Migratory |
| 3 | Emberiza citrinella | 27 | 3.5 | granivorous/insectivorous | Hedgerow-Tree | Sedentary |
| 3 | Luscinia megarhynchos | 22.5 | 4.5 | granivorous/insectivorous | Hedgerow-Tree | Migratory |
|  | **Mean** | **48.94** | **5.47** |  |  |  |
